# Leptin reverses hyperglycemia and hyperphagia in insulin deficient diabetic rats by pituitary-independent central nervous system actions

**DOI:** 10.1371/journal.pone.0184805

**Published:** 2017-11-30

**Authors:** Alexandre A. da Silva, John E. Hall, Jussara M. do Carmo

**Affiliations:** 1 Department of Physiology and Biophysics, Mississippi Center for Obesity Research, Cardiovascular-Renal Research Center, University of Mississippi Medical Center, Jackson, Mississippi, United States of America; 2 Barão de Mauá University Center, Ribeirão Preto, São Paulo, Brazil; 3 Universidade Estadual de Minas Gerais, Passos, Minas Gerais, Brazil; Stellenbosch University, SOUTH AFRICA

## Abstract

The hypothalamic-pituitary-adrenal (HPA) axis has been postulated to play a major role in mediating the antidiabetic effects of leptin. We tested if the pituitary is essential for the chronic central nervous system mediated actions of leptin on metabolic and cardiovascular function in insulin-dependent diabetic and non-diabetic rats. Male 12-week-old hypophysectomized Sprague-Dawley rats (Hypo, n = 5) were instrumented with telemetry probes for determination of mean arterial pressure (MAP) and heart rate (HR) 24-hrs/day and an intracerebroventricular (ICV) cannula was placed into the brain lateral ventricle for continuous leptin infusion. In additional groups of Hypo and control rats (n = 5/group), diabetes was induced by single injection of streptozotocin (50 mg/kg, IP). Hypo rats were lighter, had lower MAP and HR (83±4 and 317±2 vs 105±4 mmHg and 339±4 bpm), with similar caloric intake per kilogram of body weight and fasting plasma glucose levels (84±4 vs 80±4 mg/dl) compared to controls. Chronic ICV leptin infusion (7 days, 0.62 μg/hr) in non-diabetic rats reduced caloric intake and body weight (-10%) in Hypo and control rats and markedly increased HR in control rats (~25 bpm) while causing only modest HR increases in Hypo rats (8 bpm). In diabetic Hypo and control rats, leptin infusion reduced caloric intake, body weight and glucose levels (323±74 to 99±20 and 374±27 to 108±10 mg/dl), respectively; however, the effects of leptin on HR were abolished in Hypo rats. These results indicate that hypophysectomy attenuates leptin’s effect on HR regulation without altering leptin’s ability to suppress appetite or normalize glucose levels in diabetes.

## Introduction

Leptin, a peptide hormone produced by adipose tissue in proportion to the amount of body fat mass, acts in the central nervous system (CNS) to reduce appetite while increasing energy expenditure, sympathetic nerve activity (SNA), and blood pressure (BP) [1–3]. Previous studies from our laboratory and others have demonstrated that leptin is a potent regulator of glucose metabolism [4–7]. Although leptin increases insulin sensitivity in humans [8] and rodents [9], a major part of leptin’s effects on glucose metabolism is via insulin independent mechanisms as evident by the fact that chronic leptin infusion completely restores euglycemia in insulin-deficient diabetic rodents [4, 5, 7, 10]. Moreover, a major part of leptin’s chronic antidiabetic effects are mediated by direct CNS actions [4, 5, 7].

In addition to its CNS actions on body weight and glucose homeostasis, leptin also plays an important role in regulating sympathetic nervous system (SNS) activity and cardiovascular function [9, 11, 12]. Chronic intracerebroventricular (ICV) or intravenous (IV) leptin infusion not only attenuated hyperphagia and normalized blood glucose levels in insulin-deficient diabetic rats, but also completely reversed the bradycardia and restored sympathetic-vagal balance and baroreflex sensitivity [4]. However, the precise mechanisms by which leptin controls cardiovascular function in insulin-deficient diabetes and how leptin is capable of completely normalizing glucose levels even in the absence of adequate insulin production are still unclear.

Leptin regulates pituitary gland function and pituitary hormones have many important physiological functions including control of metabolic and cardiovascular functions. Some studies have suggested that reduced leptin levels in insulin-deficient diabetes may activate the hypothalamic-pituitary-adrenal (HPA) axis and that restoration of plasma leptin may attenuate hyperglycemia in large part by suppression of the HPA axis and reducing secretion of glucocorticoids [13]. However, the importance of the HPA axis in contributing to leptin’s antidiabetic action is controversial. Morton and colleagues [14] reported that glucocorticoid receptor blockade did not reverse diabetic hyperglycemia and suggested that normalization of the HPA axis and glucocorticoid signaling may not mediate the antidiabetic effects of leptin. Thus, it is still unclear whether leptin exerts its powerful CNS antidiabetic effects via modulation of the HPA axis activity or by stimulating the pituitary to release a factor with antidiabetic properties.

To unequivocally test the role of the pituitary in the CNS-mediated metabolic and cardiovascular effects of leptin without potential complications of pharmacological receptor blockade we determined whether hypophysectomy abolishes or attenuates the chronic cardiovascular and antidiabetic actions of leptin in streptozotocin (STZ)-induced diabetic rats. We found that hypophysectomy attenuated leptin’s effects to raise HR but failed to alter leptin’s chronic CNS-mediated actions to suppress food intake or to reduce blood glucose in STZ-induced diabetes in rats. These observations suggest that leptin’s actions on glucose regulation do not require intact pituitary function.

## Methods

The experimental procedures and protocols for these studies followed the National Institutes of Health *Guide for the Care and Use of Laboratory Animals* and were approved by the Institutional Animal Care and Use Committee of the University of Mississippi Medical Center.

### Animals

Male Sprague-Dawley rats weighing between 210 and 420 g were used in these experiments. The animals were kept in a temperature (23°C) and illumination (12/12 hr) controlled room. Hypophysectomized and intact rats were purchase from Charles River (Boston, Mass.).

### Surgical procedures

#### Hypophysectomy

Age-matched hypophysectomized and control rats were purchased from Charles River (http://www.criver.com/files/pdfs/surgery/hypophysectomy.aspx) weighing between 220 to 380 g. Briefly, hypophysectomy was accomplished by using the koyama’s external auditory canal method. The pituitary was removed through the ear canal with the aid of a needle using gentle suction to aspirate the gland. The rats were allowed to recover for 7 days after surgery and then shipped to our facility. Hypophysectomized rats were given 5% glucose (weight/volume) in the drinking water to provide additional caloric intake as this model is generally associated with reduced food intake. The effectiveness of hypophysectomy was confirmed by histological examination of the aspirated pituitary at euthanasia. Rats that underwent the procedure but at euthanasia were found to have intact pituitary gland were included in the control group to rule out unspecific effects of this procedure.

#### Implantation of telemetry transmitters and ICV cannula

After acclimatization to our facility for 1 to 2 weeks, the rats were anesthetized with isoflurane (2–3%) and atropine sulfate (0.1 mg/kg) was given to prevent excess airway secretion. Using aseptic techniques, a laparotomy was performed and the catheter of a pressure telemetry transmitter (Model TA11PAC40; Data Sciences International) was inserted into the abdominal aorta, distal to the kidneys, for continuous 24-hr/day blood pressure (BP) and heart rate (HR) measurements. The catheter was fixed in the aorta with a small drop of cyanoacrylate adhesive and the transmitter was secured to the abdominal wall by sutures.

After implantation of the telemetry transmitter, a stainless steel cannula (21 gauge; 10 mm long) was placed into the right lateral cerebral ventricle using coordinates previously described [15]. The guide cannula was anchored into place with three stainless steel machine screws, a metal cap, and dental acrylic, and a stylet was inserted to seal the cannula until use. During stereotaxic manipulation, anesthesia was maintained with 1.5% isoflurane. Eight days after recovery from surgery, accuracy of the cannula placement was tested by the dipsogenic response (immediate drinking of at least 5 ml of water in 10 min) to an ICV injection of 100 ng of angiotensin II.

### Hemodynamic and metabolic measurements

After recovery from anesthesia, rats were housed in individual cages for determination of daily food and water intake. The rats received food and water ad libitum during the study, and 5% sucrose in the drinking water supplementation only for hypophysectomized rats. The rats were allowed to recover for 8 to 10 days before control measurements were recorded. Mean arterial pressure (MAP) and HR were measured 24 hours/day and average values were recorded daily. BP and HR data were analyzed using Dataquest ART software (Data Sciences International). A small amount of blood (5 μl) collected from the tail snip was used to determine blood glucose levels using glucose strips (Reli On Ultima).

### Experimental protocols

To determine the role of the pituitary gland in mediating the chronic cardiovascular and antidiabetic effects of leptin, caloric intake normalized by body weight, MAP, HR and blood glucose levels were measured at baseline (control period) and during chronic ICV leptin infusion in control and hypophysectomized rats.

#### Induction of insulin-deficient diabetes

After 5 days of stable control measurements, insulin-deficient diabetes was induced by a single intraperitoneal injection of STZ (50 mg/kg,Sigma-Aldrich, dissolved in 0.5 ml of 0.05 M citrate buffer, pH 4.5, i.v.).

#### Chronic ICV leptin infusion

After 5 days of stable control measurements or 5 days after STZ injection in the diabetic groups, leptin (0.62 μg/hr at 1.0 μl/hr) was infused ICV for 7 days using osmotic minipumps (models 2001, Durect Corp.) implanted subcutaneously in the scapular region as previously described [4, 16]. We have shown that this rate of ICV leptin does not alter plasma leptin levels [4, 5].

#### Plasma insulin and glucose measurements

Blood samples were collected via tail snip after 6 h of fasting during the baseline period (day 5) and on the last day of leptin infusion (day 7) for measurement of plasma insulin by ELISA (Crystal Chem Inc) and glucose concentrations using glucose strip (ReliOn).

### Statistical analyses

The data are expressed as mean±SEM and analyzed by using 1-factor or 2-factor ANOVA with repeated measures. The Bonferroni post hoc test was used for comparisons between groups. Dunnett’s test was used for comparisons of experimental and baseline values within each group, when appropriate. Statistical significance was accepted at a level of P<0.05.

## Results

### Chronic ICV leptin infusion reduces caloric intake, blood glucose and plasma insulin levels in non-diabetic control and hypophysectomized rats

**Fig 1:**
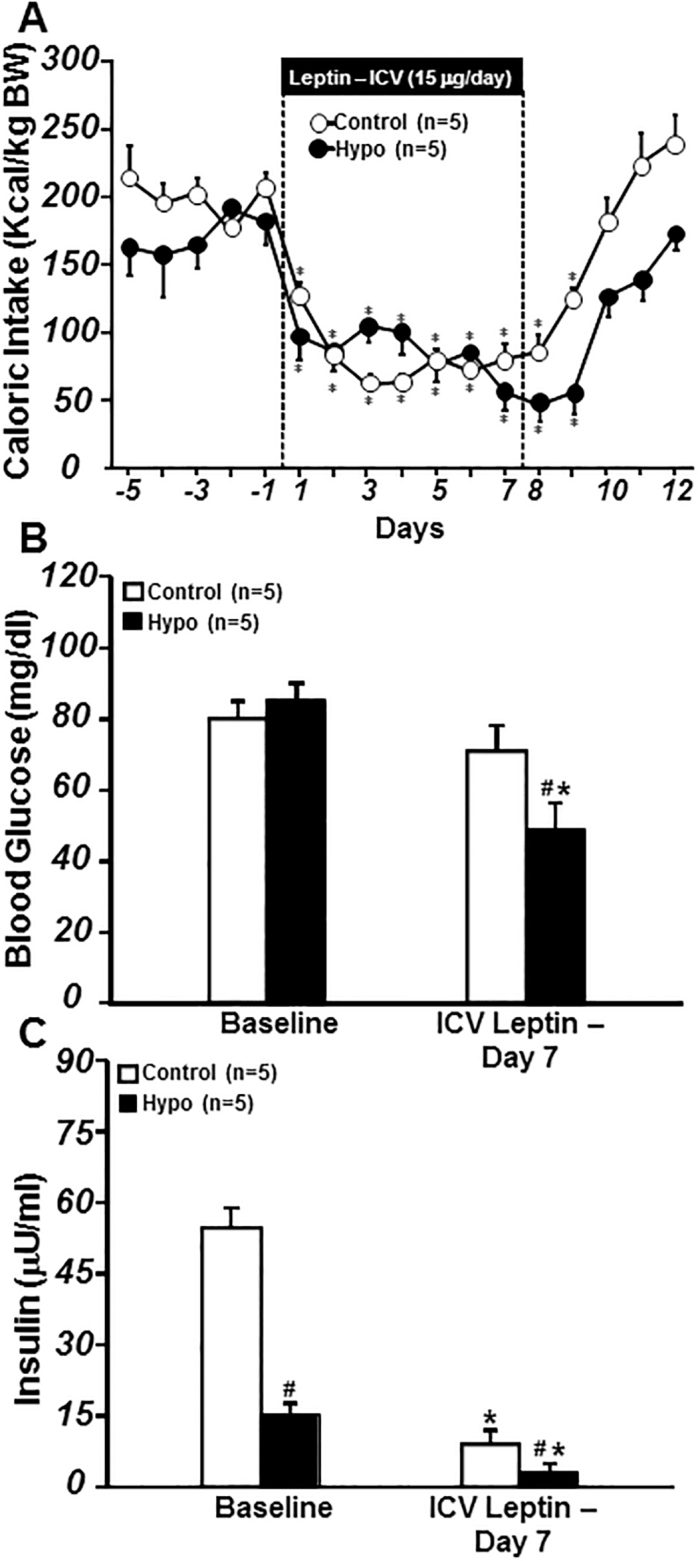
Caloric intake per kg body weight (A), blood glucose concentration (B), and plasma insulin concentration (C) responses to chronic ICV leptin infusion in control and hypophysectomized (Hypo) non-diabetic rats. Blood glucose and insulin concentrations represent values obtained on day 5 of baseline and day 7 of leptin treatment. *p<0.05 compared to baseline period; #p<0.05 compared to control group.

Despite similar caloric intake when corrected by body weight (from chow in control rats or chow plus glucose solution in hypophysectomized rats) (Fig 1A) and baseline blood glucose levels (Fig 1B), non-diabetic hypophysectomized rats had reduced body weight (215±4 vs. 415±6 g), plasma leptin levels (4.0±0.3 vs. 1.8±0.2 ng/ml), and plasma insulin levels (Fig 1C) compared to control rats. Age-matched rats were used in these experiments and the difference in body weight was likely due to the absence of the pituitary gland leading to markedly reductions of growth hormones and smaller weight gain in hypophysectomized group.

Chronic central leptin infusion markedly reduced caloric intake (Fig 1A) and body weight in control and hypophysectomized rats (415±6 to 379±6 g and 215±4 to 193±8 g for baseline and day 7 of leptin infusion, respectively). Leptin infusion also reduced blood glucose levels in hypophysectomized rats (Fig 1B) while plasma insulin levels were reduced in both groups (Fig 1C). These data indicate that hypophysectomy does not alter leptin’s ability to reduce appetite, insulin or glucose in non-diabetic rats.

### Effects of chronic ICV leptin infusion on blood pressure and heart rate in non-diabetic control and hypophysectomized rats

**Fig 2:**
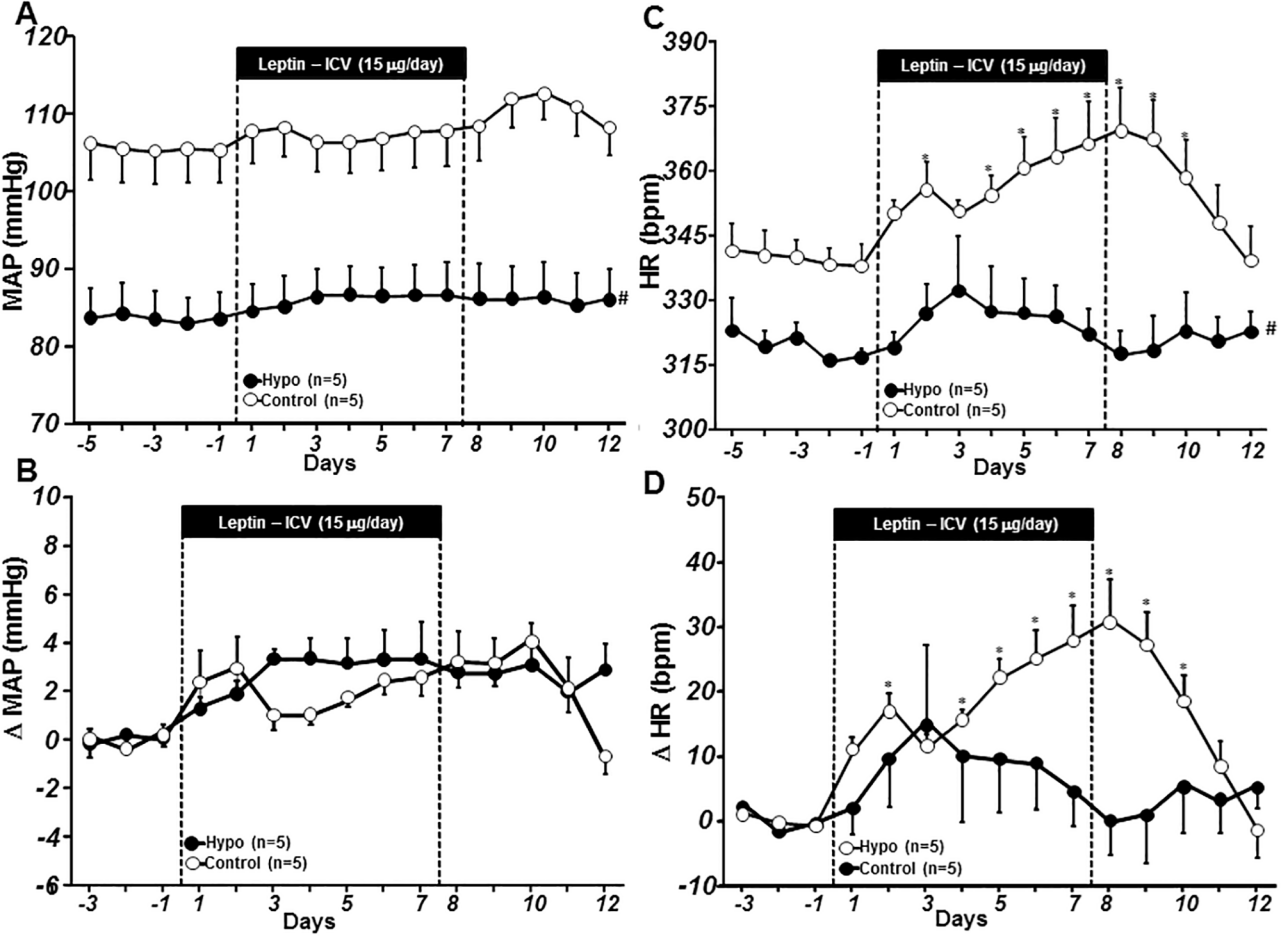
Mean arterial pressure (MAP, A), and delta MAP (B), heart rate (HR, C) and delta HR (D) responses to chronic ICV leptin infusion in ad libitum-fed control and hypophysectomized (Hypo) non-diabetic rats. *p<0.05 compared to control period; #p<0.05 compared to vehicle-treated group. Delta MAP and HR were calculated as differences between the experimental values and the average baseline values measured on the last 3 days prior to starting leptin infusion.

Non-diabetic hypophysectomized rats exhibited lower BP and HR compared to control rats (Fig 2A and 2C). Chronic central leptin infusion caused small but insignificant increases in mean arterial pressure (MAP) in control and hypophysectomized rats (Fig 2A and 2B). Leptin infusion significantly increased HR in control but not in hypophysectomized rats (Fig 2C and 2D). These results indicate that hypophysectomy markedly attenuates the chronic CNS-mediated effect of leptin to raise heart rate in non-diabetic rats.

### Chronic ICV leptin infusion reduces caloric intake and body weight, and restores euglycemia in diabetic control and hypophysectomized rats

**Fig 3:**
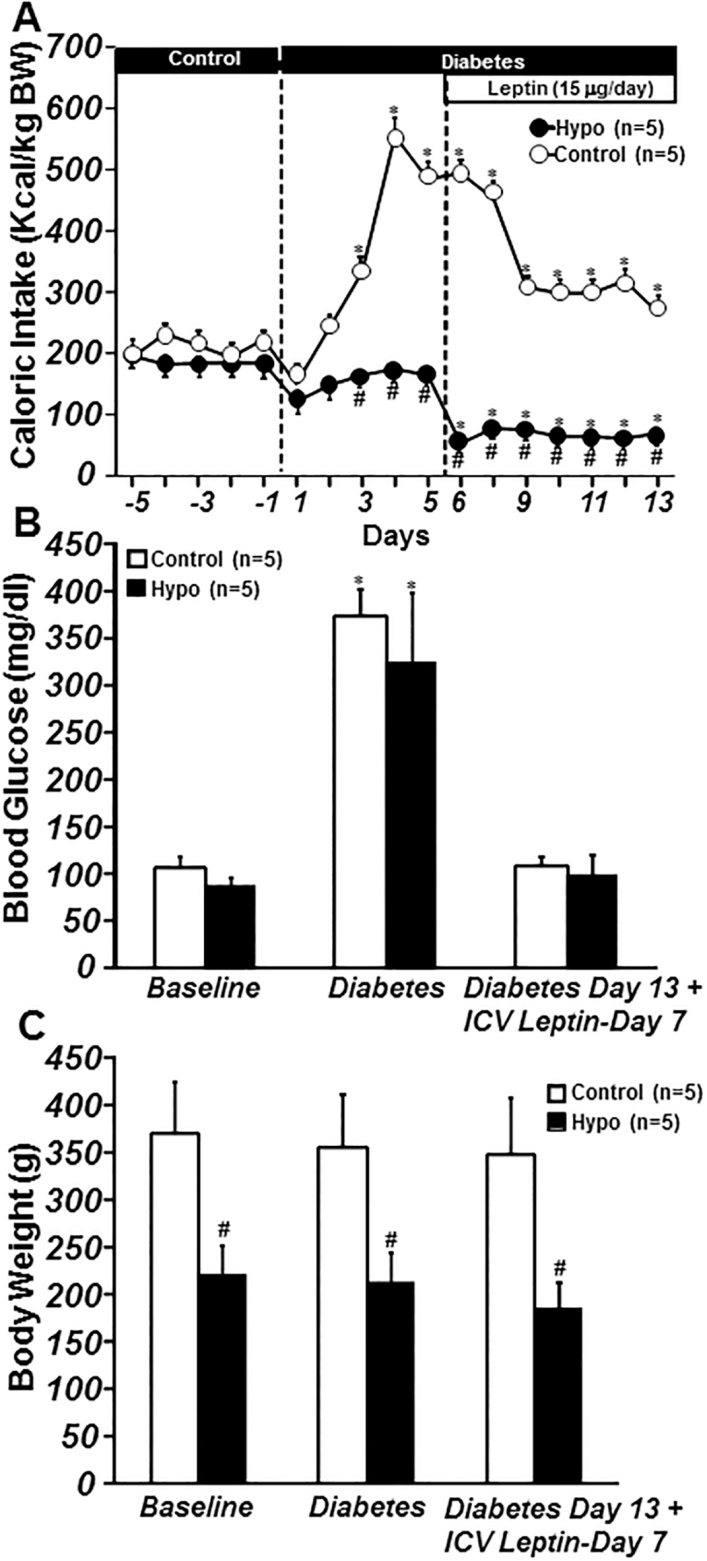
Caloric intake per kg body weight (A), blood glucose concentration (B) and body weight (C) responses to chronic ICV leptin in ad libitum-fed control and hypophysectomized (Hypo) STZ-induced diabetic rats. Blood glucose concentration and body weight represent values obtained on day 5 of baseline, day 5 post STZ injection, and day 7 of leptin treatment. *p<0.05 compared to baseline period.

Induction of insulin-deficient diabetes with STZ caused severe increases in plasma glucose (107±11 to 374±27 and 88±8 to 323±74 mg/dl) in control and hypophysectomized rats, respectively (Fig 3B), while inducing hyperphagia only in control rats (Fig 3A). Induction of STZ-diabetes also caused small but statistically insignificant weight losses in both groups (controls: 355±55 vs. 369±55 g and hypophysectomized: 212±29 vs. 220±31 g) on day 5 post STZ injection (Fig 3C). Chronic ICV leptin infusion reduced caloric intake (Fig 3A), slightly reduced body weight (Fig 3C), and returned blood glucose levels all the way back to baseline values in control as well as hypophysectomized rats (Fig 3B). These results indicate that hypophysectomy does not prevent leptin’s anorexic effects or attenuate the effects of leptin to normalize glucose levels in STZ-induced diabetic rats.

### Chronic ICV leptin infusion reverses the bradycardia induced by uncontrolled diabetes in control but not in hypophysectomized rats

**Fig 4:**
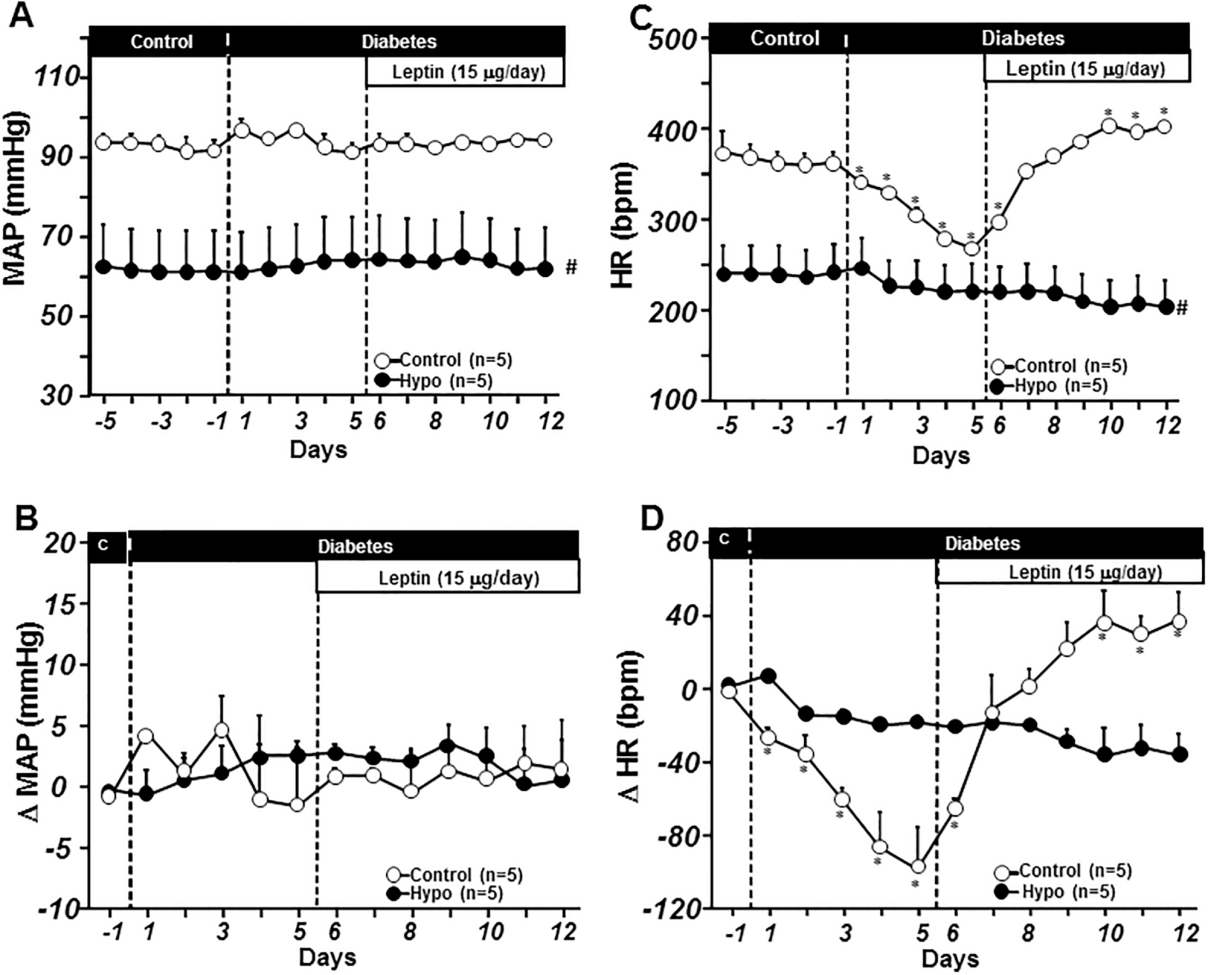
Mean arterial pressure (MAP, A), and delta MAP (B), heart rate (HR, C) and delta HR responses to chronic central leptin infusion in ad libitum-fed control and hypophysectomized (Hypo) STZ-induced diabetic rats. *p<0.05 compared to baseline control period; #p<0.05 compared control group. Delta MAP and HR were calculated as differences between the experimental values and the average baseline values measured on the last 3 days prior to STZ injection.

MAP did not change significantly during the 5 days post STZ injection (Fig 4A and 4B). However, STZ-induced diabetes was associated with bradycardia (from 369±10 to 267±3 bpm in control rats and from 241±21 to 221±18 bpm in hypophysectomized rats; Fig 4A and 4B). Chronic ICV leptin infusion for 7 days did not alter MAP in either group (Fig 4A and 4B) but completely reversed the bradycardia caused by diabetes in control rats (Fig 4C and 4D). On the other hand, leptin failed to increase HR in diabetic hypophysectomized rats (Fig 4C and 4D). These results indicate that chronic ICV leptin infusion reverses the bradycardia associated with uncontrolled type 1diabetes and that hypophysectomy abolishes the effects of leptin on heart rate regulation in this model.

## Discussion

In this study we showed that hypophysectomy did not attenuate the glucose-lowering or appetite-suppressing effects of chronic increases in CNS leptin in insulin-deficient diabetic or non-diabetic rats but did blunt leptin’s actions to increase HR. These observations suggest that modulation of pituitary function by leptin does not play an essential role in contributing to leptin’s effects on appetite or glucose regulation in diabetic or non-diabetic rats. Leptin’s chronic CNS-mediated antidiabetic actions therefore may be mediated mainly via non-pituitary factors.

Previous studies by Perry et al. [13] suggested that leptin restores euglycemia in type 1 diabetes primarily by suppression of the HPA axis and reducing glucocorticoid secretion. They observed that STZ-induced type 1 diabetic rats exhibit reduced plasma leptin levels as well as increased levels of corticosterone and adrenocorticotrophic hormone (ACTH), and that these changes were reversed by restoration of leptin levels to normal. They also reported that administration of a glucocorticoid receptor antagonist recapitulated the effect of leptin to normalize plasma glucose level in diabetic rats. Moreover, the antidiabetic effects of leptin were abolished by glucocorticoid administration [13]. These observations are consistent with the hypothesis that hyperglycemia in uncontrolled type 1 diabetes depends on activation of the HPA axis and increased glucocorticoids due to leptin deficiency.

In contrast to these findings, Morton et al. [14] showed in STZ-induced diabetic rats that neither adrenalectomy-induced glucocorticoid deficiency nor pharmacological glucocorticoid receptor blockade reduced blood glucose levels. They also found that the antidiabetic effect of ICV leptin infusion was not altered by systemic administration of corticosterone at doses that matched plasma levels in STZ-induced diabetes. These observations suggest that although leptin administration in STZ-induced diabetic rats may normalize the HPA axis, this effect cannot explain leptin’s powerful CNS-mediated glucose lowering actions.

These contrasting results led us to test directly whether the pituitary gland plays an essential role in the powerful CNS-mediated antidiabetic effects of leptin. Our observations provide unambiguous evidence that the pituitary is not essential for leptin’s chronic CNS effects that can completely normalize plasma glucose concentration in rodents with type 1 diabetes. We found that hypophysectomy, which removes all pituitary hormones, did not significantly attenuate the chronic CNS-mediated effects of leptin to reduce blood glucose in insulin-deficient diabetic rats. In addition, we found that hypophysectomy did not abolish the chronic anorexic effects of leptin. Thus, despite evidence that the acute effects of leptin on glucose regulation may be mediated, in part, by the HPA axis, our findings suggest that non-pituitary mechanisms mediate most of the chronic antidiabetic actions of leptin.

A potential contributor to leptin’s CNS-mediated glucose lowering effect is activation of the autonomic nervous system (ANS) [17, 18]. Sympathetic denervation or adrenergic blockade markedly impaired the acute CNS-mediated effects of leptin to increase glucose uptake by skeletal muscles [5, 19]. The acute effects of leptin on hepatic insulin sensitivity were also blocked by selective hepatic vagotomy [18, 20]. In contrast to these acute studies, we previously showed that chronic blockade of α_1_, β_1_, β_2_ and β_3_ adrenergic receptors did not attenuate leptin’s ability to restore euglycemia in STZ-diabetic rats [5]. We also demonstrated that neither ganglionic blockade nor hepatic vagal denervation substantially attenuated the chronic CNS-mediated antidiabetic effects of leptin [21]. Thus, although the ANS may play an important role in mediating the acute effects of leptin on glucose regulation, the chronic CNS-mediated antidiabetic effects of leptin appear to be through other mechanisms.

We previously showed that deletion of leptin receptors specifically in POMC neurons increased fasting blood glucose and insulin and abolished the reductions in blood glucose and insulin normally observed during chronic intravenous leptin infusion [22]. Also, pharmacological blockade of CNS melanocortin 3 and 4 receptors (MC3/4-R) abolished the chronic antidiabetic effects of leptin in insulin-deficient diabetic rats [23]. We also reported that POMC neuron deficiency of Src homology-2 tyrosine phosphatase (Shp2), a major intracellular signaling pathway for leptin, was associated with impaired glucose tolerance and marked attenuation of leptin’s effects to lower plasma glucose and insulin [24]. Thus, although leptin-mediated activation of POMC neurons and subsequent stimulation MC4-R play a critical role in leptin’s chronic antidiabetic effects, the factors that link these CNS effects to glucose regulation in peripheral tissues remain unclear.

We previously demonstrated that in addition to its glucose lowering effect in insulin-deficient diabetic rats, leptin also reversed several cardiovascular changes associated with uncontrolled diabetes, including marked bradycardia, reduced cardiac autonomic tone, baroreflex dysfunction and reduced intrinsic HR [4]. Furthermore, we showed that leptin’s effects on these cardiovascular parameters, except for the baroreflex, were independent of blood glucose normalization [4]. In the present study we observed that chronic ICV leptin infusion returned HR all the way back to baseline levels in type 1 diabetic rats and this effect was abolished by hypophysectomy. Previous studies have also shown that increased leptin levels cause sustained sympathetic nervous system activation in non-diabetic rodents, reversed bradycardia, and restored cardiac sympathetic-vagal balance and baroreflex sensitivity in STZ-induced diabetic rats [4].

In the current study we found that hypophysectomized rats had lower BP and HR compared to control rats and attenuated HR responses to the chronic ICV leptin infusions. Although our studies were not designed to examine the mechanisms responsible for these hemodynamic effects of hypophysectomy, reduced thyroid gland function due to low thyroid stimulating hormone (TSH) levels would tend to lower metabolic rate in many tissues and therefore tissue blood flow and cardiac output which represents the sum of blood flows to all of the tissues [25]. The low BP in hypophysectomized rats may have been caused, in part, by reduced ACTH and decreased adrenal production of aldosterone, an effect that is likely exacerbated by the fluid loss associated with uncontrolled diabetes. However, the mechanisms responsible for the effects of hypophysectomy on HR and BP regulation in diabetes and during chronic leptin infusion are uncertain and await further investigation.

In summary, we showed that the chronic CNS-mediated antidiabetic actions of leptin do not require normal pituitary function. However, leptin exerts important effects on HR regulation in insulin-dependent diabetes that require an intact pituitary. The CNS mechanisms triggered by leptin that mediate its chronic metabolic effects and that link the CNS with peripheral tissues leading to normalization of glucose levels are still unknown. Unraveling the mechanisms by which leptin regulates glucose and BP may lead to new therapeutic approaches for treatment of diabetes and other metabolic disorders as well as hypertension.

## Supporting information

S1 File Diabetes plus leptin. **Fig A**. Body weight **Fig B**. Caloric intake **Fig C**. Caloric intake/Body weight **Fig D**. Blood glucose concentration **Fig E**. Mean arterial pressure (MAP) **Fig F**. Delta MAP **Fig G**. Heart rate (HR) **Fig H**. Delta HR **Fig I**. Control plus hypophysectomized (Hypo, body weight and blood glucose).
(XLSX)

S2 File Daily data—Hypophysectomy + leptin. **Fig A**. Body weight **Fig B**. Caloric intake **Fig C**. Caloric intake/Body weight **Fig D**. Mean arterial pressure (MAP) **Fig E**. Delta MAP **Fig F**. Herat rate (HR) **Fig G**. Delta (HR) **Fig H**. MAP (day) **Fig I**. Delta MAP (day) **Fig J**. MAP (night) **Fig K**. Delta MAP (night) **Fig L**. HR (day) **Fig M**. Delta HR (day) **Fig N**. HR (night) **Fig O**. Delta HR (night) **Fig P**. Blood glucose—diabetes.
(XLSX)
